# Sensor Fusion of a Mobile Device to Control and Acquire Videos or Images of Coffee Branches and for Georeferencing Trees

**DOI:** 10.3390/s17040786

**Published:** 2017-04-06

**Authors:** Paula Jimena Ramos Giraldo, Álvaro Guerrero Aguirre, Carlos Mario Muñoz, Flavio Augusto Prieto, Carlos Eugenio Oliveros

**Affiliations:** 1 Centro Nacional de Investigaciones de Café, Manizales 170009, Colombia; alvaro.guerrero@cafedecolombia.com (Á.G.A.); carlos.oliveros@cafedecolombia.com (C.E.O.); 2 Universidad Nacional de Colombia, Bogotá 11001, Colombia; carmmunozper@unal.edu.co (C.M.M.); faprietoo@unal.edu.co (F.A.P.)

**Keywords:** sensor fusion, precision agriculture, machine vision, smartphone, image quality, field conditions, coffee plantation

## Abstract

Smartphones show potential for controlling and monitoring variables in agriculture. Their processing capacity, instrumentation, connectivity, low cost, and accessibility allow farmers (among other users in rural areas) to operate them easily with applications adjusted to their specific needs. In this investigation, the integration of inertial sensors, a GPS, and a camera are presented for the monitoring of a coffee crop. An Android-based application was developed with two operating modes: (*i*) Navigation: for georeferencing trees, which can be as close as 0.5 m from each other; and (*ii*) Acquisition: control of video acquisition, based on the movement of the mobile device over a branch, and measurement of image quality, using clarity indexes to select the most appropriate frames for application in future processes. The integration of inertial sensors in navigation mode, shows a mean relative error of ±0.15 m, and total error ±5.15 m. In acquisition mode, the system correctly identifies the beginning and end of mobile phone movement in 99% of cases, and image quality is determined by means of a sharpness factor which measures blurriness. With the developed system, it will be possible to obtain georeferenced information about coffee trees, such as their production, nutritional state, and presence of plagues or diseases.

## 1. Introduction

Precision agriculture (PA) emerged from the need to correctly program activities on a farm, to make efficient use of economic, human, and technological resources, to reduce environmental impact, and to make crops traceable. The need to make decisions on a plantation, based on real crop measurements (which are referenced terms of in space and time) has resulted in the development of different technologies to estimate, evaluate, and understand variables on plantations that affect production. PA works, along with the measurement of multiple variables in field, which require computational systems for their storage and processing. Some devices have been developed specifically for the measurement of nutritional stage [1], leaf area index [2], and soil analysis [3], which, due to their high cost, are not accessible to small agriculturalists. Nowadays, mobile devices are computers with multiple sensors, control and variable-monitoring capacities, and can be used under field conditions using applications adjusted to the needs of agriculturalists, who already have this type of technology at hand.

### 1.1. Related Work

Mobile devices are used in agriculture mainly to control drones [4] or vehicles on land [5], to create ways to store information [6], or to connect to a network of wireless sensors, in order to manage, store, and upload each one of the measurements to the cloud in real time [7]. However, mobile devices work as tools for acquisition and processing of information, as well as, control and monitoring of the mentioned information. This is true, for example, in the measurement of chlorophyll by means of built-in camera and accessories used to improve the quality of the images to be acquired [8], or the measurement of the vigor and the porosity of the canopy in the vine [9] in order to track plant growth.

One of the main techniques that is being used nowadays in precision agriculture is machine vision and, with it, different types of cameras, adjusted to work in environments under uncontrolled conditions [10,11,12,13,14,15,16,17,18]. Depending on the type of processing technique, computational requirements differ, and vary according to the size of the images, the number of channels per scene, three-dimensional processing [16,17,18] or two-dimensional processing [10,11,12,13,14,15]. Most of the applications for mobile devices created for image processing are run on simple processes such as binarization and pixel count [19]. In other cases, the images are uploaded to a server for future processing [20]. In the case of the latter, it is necessary to use a sufficient number of high-quality images to be uploaded to the cloud, without duplicating information or saturating the databases. The resolution of images taken in field conditions represents a critical aspect, since the lighting and sharpness conditions, as well as the contrast and occlusion of vegetative structures can affect the performance of implemented vision algorithms.

By using georeferencing sensors such as a low-cost GPS, it is possible to relate a location mark to the acquired information, using any type of device. Nonetheless, the error in georeferencing can range from ±2.5 m to ±5 m, a distance that may, many times, be greater than the planting distance between trees in some crops, although, in plantations with large areas, it is usually sufficient to obtain information about their state [21]. In other cases, information from the GPS is insufficient, and must be corrected by means of an Inertial Measurement Unit (IMU), where the direction of movement angles are used to create three-dimensional maps [22]. The use of mobile devices can integrate GPS and IMU systems, so as to balance out position errors through different information integration intervals, thus showing errors of up to 14 cm [23] in indoor, controlled environments.

For applications in agriculture, acquisition control, image selection, and georeferencing with higher resolution can be attained through the use of mobile devices with built-in sensors. Nowadays, most of the mobile applications with sensors for agricultural use, employ GPS and cameras [24]. That said, it is possible to integrate the inertial sensors in mobile phones in order to control image acquisition and to georeference trees that have fewer errors in outdoor working environments and in uncontrolled environments.

### 1.2. The Problem and the Contributions of This Investigation

In a coffee crop, it is important that plant health and nutritional states be constantly evaluated, and that decisions be made, which impact production, costs, and quality. Nonetheless, and in spite of its world-wide importance, the use of technologies for such aims is minimal, and the workforce hired does not always have sufficient agronomic knowledge of the coffee crop. Unlike other technified crops, coffee is sown on small parcels with a high sowing density (<5000 plants/ha), and, in some cases, in mountainous, steep terrains (~50%) [25], which makes it difficult to use technology or machinery. In this paper, the use of sensors built into mobile devices to monitor coffee crops is described.

Based on the images acquired of coffee branches, it is possible to obtain information about a plant, its production, nutritional aspects, and presence of plagues or diseases. The acquisition of these images is complex, as space is limited, and it is not possible to obtain a single image per branch; it is necessary that images be taken successively to obtain all necessary information. Additionally, it is necessary to georeference images acquired of each tree, however the mobile device’s GPS does not have the resolution necessary to adequately georeference each one, since georeferencing errors in this system (±2.5 m and ±5 m) are greater than the distance between plants, a minimum of 0.5 m.

In this article, the integration of a mobile device’s inertial sensors, GPS, and camera for acquisition control and the selection of images of coffee branches, as well as the georeferencing of each tree with errors of less than 0.5 m for uncontrolled conditions in outdoor environments is presented.

The contributions of this study are: (*i*) a mobile application for the control of image acquisition, quality measurement, automatic selection of the most suitable images, and definition of the location mark for each tree; (*ii*) an inertial navigation system for measuring displacements at low velocities, using an initial value delivered by the mobile device’s GPS as a point of departure; (*iii*) an inertial navigation system for control of image acquisition as the device moves over the branch at low velocities; (*iv*) a system to measure image quality, by means of sharpness indexes for the selection of frames from the acquired videos, which are related to the mobile device’s rate of speed during acquisition. All this to equip the farmer with a low cost, easily-accessed, mobile tool, which permits them to collect georeferenced information about their crop, with relative accuracy of ±15 cm, and which permit complete mobility on the plot. 

This paper has the following structure: Section 2 presents the image acquisition system of coffee branches in field conditions. The inertial navigation system (both for image acquisition control and georeferencing with higher resolution) is shown in Section 3. The set-up and evaluation of integrated sensors are presented in Section 4. Finally, the conclusions, projections, and future work are shown in Section 5.

## 2. Image Acquisition of Coffee Branches in Field Conditions with a Mobile Device

**Figure 1:**
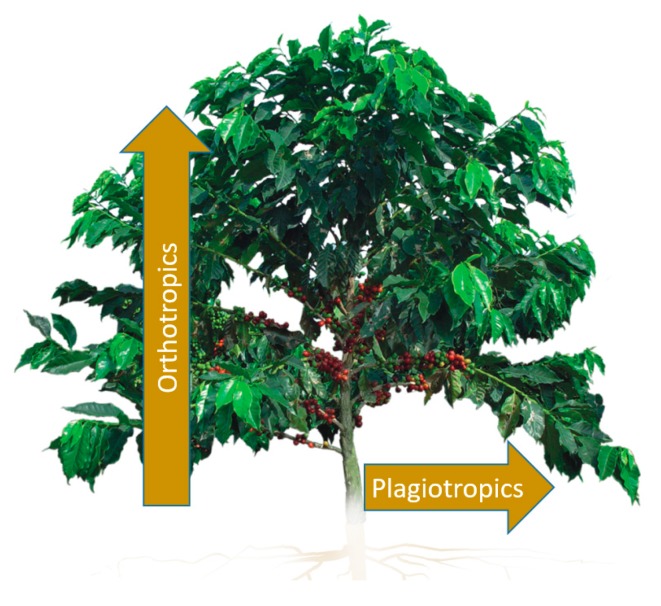
Morphological aspects related to coffee tree branches.

Technified coffee crops are densely planted areas with 5000 to 10,000 trees per hectare. Such crops can be found in tropical and subtropical zones of Asia, Oceania and the Americas. In the tropical zones of the Americas, there are mountainous regions, ranging from 1200 to 2000 meters above sea level, with specific climate and soil conditions which produce high-quality coffee. Coffee trees belong to the Rubiaceae family. They are known for their leaves, which grow in pairs, without divisions or smooth edges, their hermaphroditic flowers, and their fruit, which usually contains two seeds. The most noticeable morphological aspects are related to the two types of stems, Figure 1: (*i*) Orthotropics: the main stem grows vertically; (*ii*) Plagiotropics: the main, secondary, and tertiary branches grow horizontally. Coffee plants may grow up to 2.8 m in technified crops and, on average, produce 24 branches per year, although this varies according the climate and soil conditions of each crop. The production of coffee takes place on the plagiotropic branches, mainly in the middle third of the tree, which moves as the tree grows. 

Coffee production can be estimated based on the fruit count on productive branches [26,27,28]. Currently, destructive samplings are carried out to estimate the production of trees and parcels. However, with image processing, it is possible to detect, classify, and count fruits on branches, and at the same time, obtain estimation models that relate automatically measured fruits to real observed fruits on each of the branches, with no need for destructive samplings. In hopes of using mobile devices, machine vision, and information and communications technology (ICT) to measure the production of a coffee tree, an integral system was developed so as to acquire images of coffee branches, and detect the movement of the device over the branch, in order to control image acquisition, and to detect movements in the crop, so that each of the sampled trees may be georeferenced.

### 2.1. Conditions for Image Acquisition in the Field

The images to be acquired are horizontally oriented and correspond to coffee branches which are present in the middle third of a tree, which means that the information to be acquired can be found in the foliage of a tree. This condition, added to the height of the trees, which can be in excess of 2 m, as well as the very steep terrain, hinders image acquisition. There is no lighting control and the background of the images corresponds to other branches of the same or of adjacent trees. Additionally, due to the aforementioned morphological conditions, it is not possible to take a photo of an entire branch due to the minimal distance between branches. The alternative is to take a set of images that represent the entire branch, viewed from above. That is to say, there will only be images obtained from a single side of the branch. It is possible to carry out this task using a mobile device’s camera or by taking a video of the branch. In either case, the images must be taken in adequate conditions in order to be processed.

The images of the coffee branches must be homogeneous. Branch information must be in the center of the horizontally oriented image and at least a 70% of the image must correspond to the checked branch. Images may have lighting issues generated by self-shadowing or excess of solar radiation on the branches. These problems cannot be controlled, and a particular time of day must be selected for image acquisition, in order to avoid them. On the other hand, all blurry and unfocused images must also be avoided. In order to acquire images in the best conditions, the following strategies were implemented: (*i*) to avoid defocusing: guarantee distance between the branch and the camera, and (*ii*) to avoid blurring: measure the displacement velocity and guarantee acquisition at low velocities.

When acquiring information in field conditions, it becomes difficult to control the touch screen of the mobile device, as contact of any object with the screen, such as a leaf or a drop of water, may change acquisition conditions: (*i*) focus on a different angle in the image; (*ii*) zoom in and out; (*iii*) stop a recording or the accidental capture of images. This becomes even more critical if the following aspects are taken into account: (*i*) the short distance between branches; (*ii*) the probability that leaves from nearby branches touch the screen; or (*iii*) damp coffee plantations, as a consequence of the rainy season. Additionally, times of day with too much sunlight make it impossible to properly interact with the screen of the mobile device.

### 2.2. Design Specifications for Image Acquisition

The developed application and its accessories must take into account each one of the conditions of image acquisition. For this reason, the following design specifications were generated. 

#### 2.2.1. Camera Configuration for Images/Videos

Images were acquired with the main camera of a Samsung Galaxy S5 SM-G900M mobile device (Seoul, South Korea). The camera was configured in Full-HD (1920 × 1080), 30 fps, without activating the flash. The white balance and ISO were set on automatic, and the exposure value on 0. The remaining characteristics had the default configuration set by the mobile device. Other image acquisition modes, such as burst mode, were not viable, since the on-screen preview was lost as the phone was moved along the branch.

#### 2.2.2. A Mobile Device Holder: Avoiding Image Blurriness

**Figure 2:**
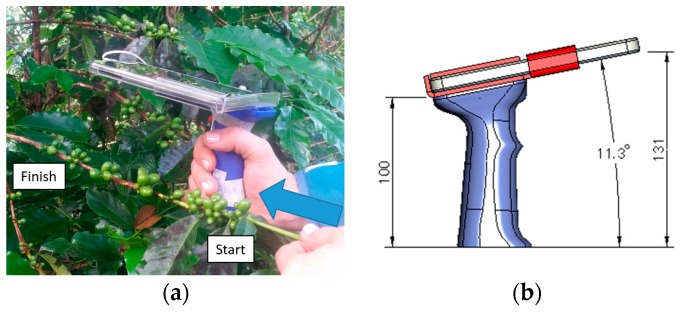
Movement and mobile device holder over the coffee branch. (**a**) Movement of the mobile device parallel to the branch; (**b**) Dimensions of the mobile device holder.

The images of a coffee branch were acquired as the mobile device moved in parallel over the branch, but only the fruits on one side of the branch were photographed (Figure 2a). With the aim of guaranteeing a constant distance between the camera and the branch, a holder was designed, with a handle, a secondary base to hold the device, and a pair of buttons to control focus and image acquisition (Figure 2b). An angle of 11.3° guaranteed that the user can see and control the branch focus before initiating the video recording. This angle could change as the video was recorded, since the user may have adjusted the mobile phone’s position, if the branch changed shape or curved. If the base of the holder is aligned with the longitudinal axis of the branch, a distance of between 80 and 150 mm is guaranteed between the camera and the branch, which is sufficient for information from one part of the branch to be contained in the image acquired by the mobile device.

#### 2.2.3. Focus Control and Image Acquisition Using a Push-Button

Because the screen was not used as an interface, the handle described in Section 2.2.2 had two buttons integrated, the first to focus the central zone of the image, and the second to start/stop the recording of videos for acquiring static images, depending on the initial setting chosen. Lighting conditions, branch contrast, and fruit occlusion were not controlled and, as previously mentioned, the mobile device was activated without using flash. Through use of the holder and the interface, by means of the buttons on the handle, there was complete control in the acquisition of branch images. 

#### 2.2.4. Measurement of Movement in the Acquisition of Videos/Images and Calculation of Blurriness Caused by Movement

In preliminary tests, it was determined that it was necessary to have wait time between initiation of filming and the filming of the length of the branch, in order for the branch to be in focus, and to wait for the other sensors employed to respond. For this reason, the beginning of the video does not correspond with the beginning of movement, and an analysis of displacement generated over the branch is required to segment the video exclusively into moments of movement. Also, it is important to consider that the speed of movement had to be low, as the camera had a 30 fps frequency, and lighting conditions were uncontrolled, two aspects which could easily cause blurriness. For this reason, inertial sensors were integrated, so as to detect the beginning and end of movement. With this information, it was possible to segment the videos into moments when the device was moving, and it was easily determined whether the video or images should be acquired once more or were suitable. Additionally, a blurriness index was established, which indicated whether or not there were movement problems. Likewise, a blurriness rate was instituted, with which it was possible to infer the displacement velocity that was required to obtain image information. This process is explained in Section 4. On average, movements over productive coffee branches had a duration of 15 s when recordings were made at a speed of 3 cm·s^−1^ and spaced at 50 cm. Section 4 explains how the measurement of the beginning/end of the movement times were taken, through the detection of the angular velocity at slow speeds over the branch, to select the part of the video to be processed. 

#### 2.2.5. Types of Acquired Images

**Figure 3:**
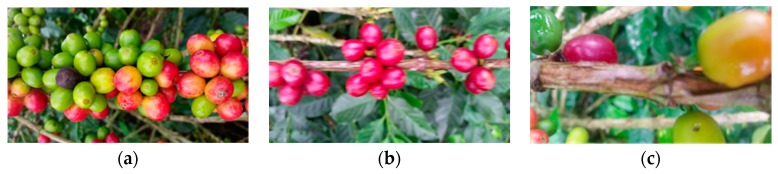
Types of acquired images. (**a**) Ideal image, well-focused, centered, and without blurriness due to movement; (**b**) Image blurred by movement; (**c**) Unfocused image, due to short distance between camera and branch.

The information was acquired under uncontrolled field conditions. Videos were filmed over a period of days, and at different times of day, in order to guarantee different lighting conditions. Lighting was in accordance with present climatic conditions at each time of information collection. In the background of images, there is soil, weeds, dry leaves, and branches from other trees. A single branch may measure between 40 and 60 cm, so when making a video of said branch with a 30 fps camera, at an average velocity of 3 cm·s^−1^ (to avoid blurriness), approximately 450 frames were obtained. Of these, only 20% are necessary in order to apply 2D and 3D detection algorithms [29]. Some examples of the types of images acquired can be seen in Figure 3. Figure 3a shows the images chosen for post-processing and Figure 3b,c show blurry and unfocused images, which must be avoided.

## 3. Inertial Navigation System (INS)

Measuring movement at low velocities is a difficult problem to resolve by means of inertial sensors such as the accelerometer. For a gyroscope, the measurement of small movements is possible, whenever the movement is rotational. In this case, the movement generated over the branch is of a linear character, and so cannot be precisely measured by an accelerometer, due to the low speed, or with a gyroscope, due to its linear character, although it may be detected by the latter. Additionally, georeferencing a tree on a high-density plantation with the GPS on a mobile device poses a number of complications, as the only localization provider in rural areas, in this study, were satellites available from the coffee farm. In rural areas, the localization provider has no connection to internet or mobile network, and the following problems may be generated: (*i*) GPS response time: in the time that a sensor sends a single coordinate, several trees on the lot have been examined, and so the journey is wasted (*ii*) resolution of georeferenced points; GPS resolution on a mobile device may be between 2.5 and 5 m, while the plantation has trees planted every 0.5 m; (*iii*) GPS precision: during the navigation of a lot, GPS readings are neither constant nor repeatable. In this chapter, a way to use inertial sensors to control image acquisition in order to adjust initial GPS readings to georeference each of the trees checked, and obtain precision in terms of centimeters during the tour of a coffee plantation is proposed.

**Figure 4:**
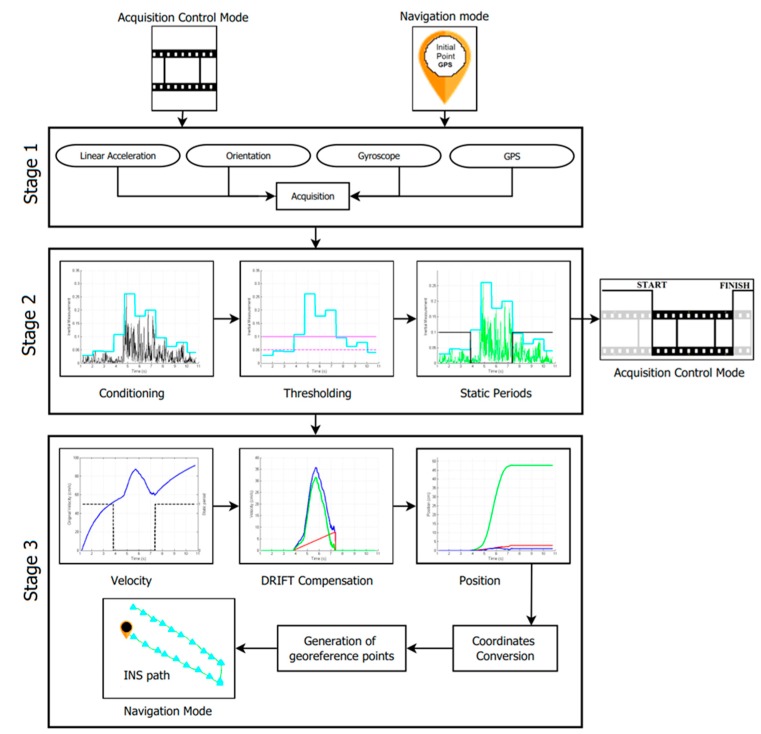
Inertial Navigation System (INS) proposed. Diagram of the processing of inertial signals acquired from the mobile device, for each of the two modes of operation.

An Inertial Navigation System (INS) was developed to detect and measure movement, in centimeters at low velocities, for navigation on plantations, and for control of image acquisition, Figure 4. In this section, each of the steps followed in order to obtain said movement measurements are explained. Stage one refers to the acquisition of inertial signals, stage two consists of the preprocessing of these signals, in order to obtain movement periods, and stage three consists of the calculation of velocity and displacement of generated movement. 

### 3.1. Capture and Analysis of Movement

Through use of an inertial measurement unit, it is possible to capture and analyze movement through the integration of linear accelerations, angular velocity, and the orientation of accelerometers, gyroscopes, and magnetometers respectively. The information obtained with each sensor is integrated in such a way that velocity, position, and direction can be determined with the least possible number of errors. If the initial location details from the GPS are added to this information, it is possible to obtain high-resolution georeferenced navigation data , because, while the GPS error ranges from ±2.5 m to ±5 m, the error in the captured movements with an inertial measurement unit can be organized in centimeters. In this section of the paper, the Inertial Navigation System is presented in order to detect and measure movements in centimeters, at low velocities. Movement information is acquired with a mobile device’s inertial sensors, and with it, the quality of the acquired images is controlled by the camera of the same device. Later, selected frames from these videos are chosen, and the information for each tree is georeferenced. To achieve this result, an algorithm for the processing of inertial information was developed.

### 3.2. Proposed Inertial Navigation System (INS)

The developed algorithm has two operating modes to analyze inertial information in the field: (*i*) Navigation Mode (NM), with which it is possible to georeference each one of the coffee trees to be checked on a plantation with centimeter-based resolutions, and (*ii*) image Acquisition Control Mode (ACM), where the operation detects the movement of a mobile device, at low velocities, over a coffee branch, and controls image acquisition. In the first operating mode, the system uses linear acceleration, angles, and GPS direction information. In the second operating mode, the system uses angular velocity and linear acceleration information. In both cases, the acceleration signal (in Navigation Mode—NM) and the angular velocity signal (in Acquisition Control Mode—AMC) are be taken as inertial signals (*is*) and are used to detect or measure movement; some variables depend on the operating mode.

The algorithm developed proposes a new technique to find movement periods through the detection of local maxima on the previously defined inertial signals. For each one of the two modes, linear acceleration information is used with the purpose of finding velocities and displacements. Cumulative errors are eliminated in two ways: (*i*) Elimination of static periods, and (*ii*) compensation for the DRIFT (deviation of the measure). Correct movement detection is generated in 98% of the cases. Additionally, this technique uses orientation values to orient the movement, with respect to the magnetic north in the NM mode; in the ACM mode, such orientation is not necessary, because detection of whether there is movement or not is the only requirement. The proposed system is divided into three stages: (*i*) acquisition and conditioning of sensor signals available on the mobile device; (*ii*) determination of static and movement periods; and (*iii*) calculation of velocity and displacement, with compensation of the DRIFT. Figure 4 shows a diagram of the proposed system.

#### 3.2.1. Acquisition and Conditioning of Signals

The mobile device used has a set of movement sensors (Table 1), which delivers signals previously measured and conditioned to face likely problems, such as interference due to temperature, and the effect of gravity. The system is configured to create a text file with information from the linear acceleration sensors (3 axes), gyroscope (3 axes), the three orientation triangles (yaw, pitch, and roll), information from the magnetometer, and the longitude and latitude data from the GPS. The system creates two independent files (one for each mode), but the two of them with the same structure and information. The NM mode file contains the entire displacement per parcel, and the ACM mode file has one file for each one of the scanned coffee branches. The sampling time is 10 milliseconds in both modes.

#### 3.2.2. Static Periods

The mobile device is taken into the field by an operator, who grips the holder described in Section 2. The movements generated by the operator have two objectives: (*i*) measure the distance covered by the operator on the lot (NM); and (*ii*) determine the moment of initiation and finalization of the operator’s movement over the branch (ACM). Two types of movement are identified*: (i*) idle: the device is held by the operator, without moving, but the values acquired by the sensors are not equal to zero; and (*ii*) in movement: the operator moves the device over the branches or moves around the plantation, while the corresponding signals are acquired. In theory, when the system is without movement (idle), the linear acceleration reading should be zero. However, this is not the case—measurements of ±10 mg have been obtained. Additionally, ±1 mg calibration errors can occur, which generate a 1.66 m·s^−1^ error [30]. In this investigation, the ±10 mg readings, when the device is held by the operator, without generating movement, can cause a 16.66 m·s^−1^ error [30]. As a result, a calibration process is necessary, to determine as of what values the device is truly moved by the operator, and to create a strategy to force the accelerations found below real movement to zero, and thus avoid cumulative errors in position measurements.

**Figure 5:**
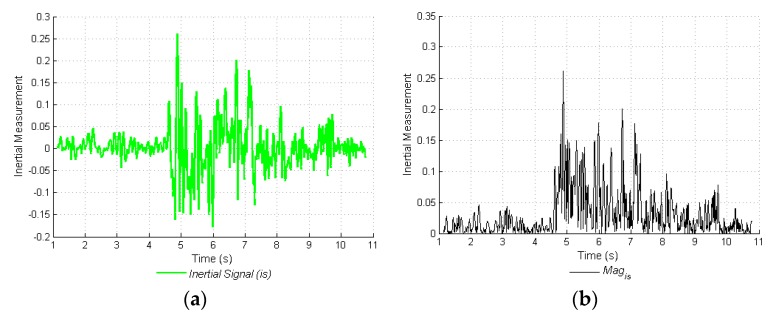

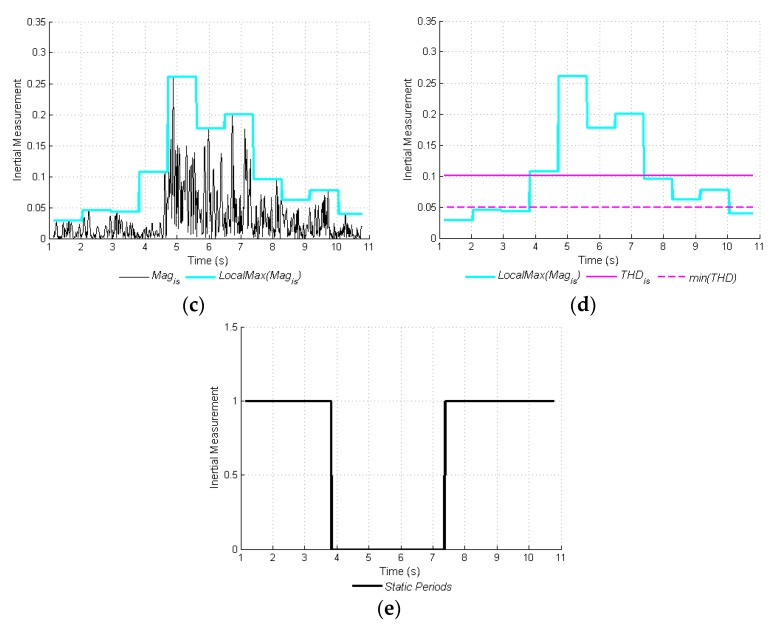
Calculation of static periods. (**a**) Raw inertial signal; (**b**) Magnitude of the inertial signal; (**c**) Local maxima of the signal; (**d**) Minimal and dynamic applied threshold; (**e**) Vector of static periods.

In order to determine static periods and those of movement, the magnitude of the inertial signal is used (*Mag_is_*, Equation (1)), which is adequate for each mode (Figure 5a). The magnitude of the inertial signal (*is*) corresponds to: (*i*) the linear acceleration in axis *X* (*AccL_x_*) and the linear acceleration in axis *Y* (*AccL_y_*) in the NM mode; and (*ii*) the angular velocity in axis *Y* (ω*_y_*) in the ACM mode (Figure 5b). The value of such magnitude changes considerably with even the slightest alteration (Equation (1)):

(1) $$Mag_{is}(t)=\sqrt{is^{2}}∴is:\left\{AccL_{x};AccL_{y};\omega_{y}\right\},$$

In either of the two modes, the estimation of local maxima on the magnitude of the inertial signal is applied. The calculation of the local maxima (*LocalMax(Mag_is_)*, (Equation (2)), consists of dividing the magnitude signal into fixed intervals of *M* samples and calculating the maximum value of the magnitude in each interval (*j*). All of the magnitude signal values take the maximum value of that interval, thus obtaining the signal in Figure 5c. The previously mentioned value, *M,* was obtained through a statistical evaluation, and corresponds to 85 samples:

(2) $$LocalMax\left(Mag_{is}\right)\left(j:j+M\right)={\max\left(Mag_{is}\right)|}_{j}^{j+M}∴j=0,M,2M...t,$$

In order to obtain movement periods, the local signal maximum is calculated, and compared with a dynamic threshold (*THD_is_*, Equation (3)), which is calculated with the mean (*μ*(*Mag_is_*)) and the standard deviation (*σ*(*Mag_is_*)) of each magnitude, as well as an adjustment factor, *K,* which depends on each inertial sensor used.

(3) $$THD_{is}=\mu \left(Mag_{is}\right)+K\cdot \sigma \left(Mag_{is}\right),$$

There is a minimum threshold value, which corresponds to the maximum magnitude value of the inertial signal, when the device is steady (Figure 5d). This minimum threshold value is obtained through an inertial signal characterization, when the operator has the device in hand, but generates no movement. The dynamic threshold can change, depending on the person holding said device, and so should be measured for each operator. In Table 2 both the *K* values and the minimal threshold for each mode may be observed. 

Finally, a Boolean vector is determined, where there are zeros in the movement periods and ones in the static periods (Figure 5e). This vector acts to force sensor signals to zero when the device is idle, and to leave signals unmodified when a movement is detected, which eliminates cumulative errors. With the information obtained from periods of rest and movement, videos acquired from movement over coffee branches are segmented; the ACM mode finalizes its process in this section of the INS proposal.

#### 3.2.3. Velocity and Displacement, Compensation of the DRIFT

**Figure 6:**
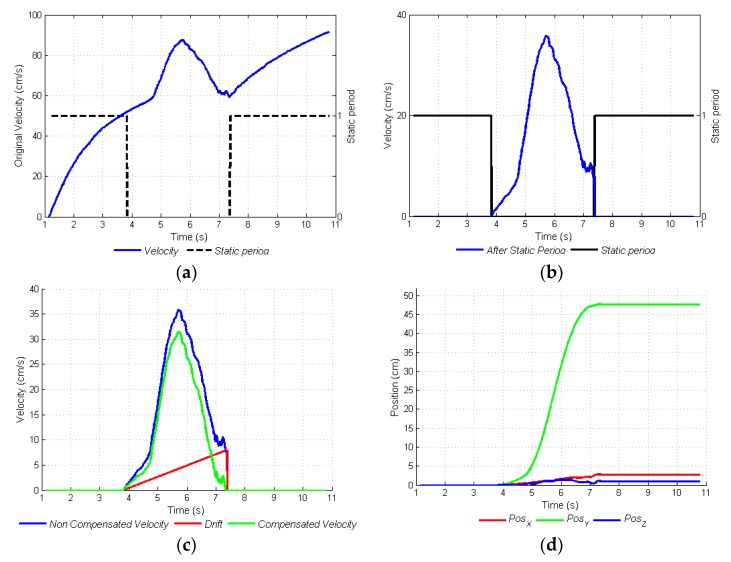
Adjustment and calculation of velocity and position of movement. (**a**) Velocity without adjustments; (**b**) Adjusted velocity for static periods; (**c**) Compensation for the DRIFT; (**d**) Position signal obtained when integrating compensated velocity.

To measure velocity, the next step is to integrate the linear acceleration to the two operating modes with respect to the sampling time (10 ms). In Figure 6a, the calculated velocity is shown (in blue), as is the increasing effect of the velocity generated by cumulative error in stationary periods. In Figure 6b, we observe that that the velocity signal is equal to zero in the static periods. Thus, the aforementioned cumulative error is eliminated. Yet, when the movement ends, the final velocity has a value different than zero. This is solved by means of the estimation and elimination of DRIFT. DRIFT is the deviation generated by the same cumulative error in the velocity signal. Although DRIFT is generated by acceleration, it can be estimated, considering that the final velocity and movement period are known. This estimation corresponds to a straight line which passes through the initial and final velocity points, during this period. The straight line generated by Equation (4) shows the rate of change (*m*) generated by DRIFT, in the velocity of a time t (stationary period), and should be eliminated to obtain a final velocity of zero, and so eliminate the cumulative error of DRIFT in the velocity signal, during the period of movement, as shown in Equation (5). In Figure 6c non-compensated velocity, estimated DRIFT, and compensated velocity are shown.

(4) $$\hat{\varepsilon }=m\cdot t,$$

(5) $$vel_{compensated}=vel_{non-compensated}-\hat{\varepsilon },$$

In Figure 6d, the 50 cm displacement can be seen, which results from the integration of the compensated velocity signal from Figure 6c. This signal corresponds to a relative position acquired with the INS during a displacement in navigation mode. During the tour of the plantation, there are a number of stationary periods, as well as those with movement. In each period of movement, localization coordinates are updated, from the movement generated in the *X* and *Y* axes, and the orientation measured by the mobile device (*Azimut*). It is necessary to emphasize that the first georeferenced position comes from the GPS, but the following coordinates are generated by the INS.

The starting point obtained with the GPS is given in ellipsoidal coordinates [Latitude (Lat), Longitude (Lon)] using the world geography referencing system WGS84. The INS proposed delivers the position in meters (*X* and *Y* axes), which could require coordinate conversion to integrate both measurements in meters. The conversion between ellipsoidal coordinates [Lat, Lon] and Gauss-Krüger plane coordinates [North (N), East (E)] [31] is performed, in order to move the entire georeferencing system to Gauss-Krüger plane coordinates and take movement information from the INS. Later, the inverse conversion is performed, to take the information to a georeferenced system [Lat, Lon] and put it on a map in Google Maps.

In relation to movement direction, the ellipsoidal coordinate system and that of Gauss-Krüger plane coordinates are directed toward geographical north. The mobile device gives the direction referencing magnetic north. Due to this, a compensation is made, in magnetic declination (MD), which, for the zone where the work was carried out, is −6.13°.

After calculating the displacement of the X and Y axes in the mobile device, and obtaining the adjusted orientation, it is necessary to calculate the new georeferencing coordinate once more. Equation (6) shows the calculation of the adjusted direction, with magnetic declination. Equation (7) shows the relative displacement of the X and Y axes, measured with the mobile device, and the INS in Gauss-Krüger plane coordinates [N, E]. Equation (8) is the current position, saving the information prior to the path. The initial values held by N and E correspond to the initial GPS reading in Gauss-Krüger plane coordinates. 

In Equation (6), θ is the angle of direction with the magnetic declination correction. In Equation (7), ΔN, ΔE, ΔX, ΔY are the changes in the N and E coordinates, and in the *X* and *Y* displacements, respectively:

(6) θ=Azimuth−DM,

(7) ΔN=ΔY∗cos(θ)+ ΔX∗cos(θ+90),ΔE=ΔY∗sin(θ)+ ΔX∗sin(θ+90),

(8) N=N+ΔN,E=E+ΔE,

The position measured with the INS is relative to the initial point of movement. The initial point is georeferenced using the mobile device’s GPS, and may have localization errors between ±2.5 m and ±5 m. The INS developed generates routes which avoid those GPS complications mentioned at the beginning of this section, with more precise displacement measurements, and adjusted in the georeferenced locations.

## 4. Results

The algorithm described in Section 3 was calibrated for navigation mode, and evaluated for both modes. In navigation mode, the measurement of displacement on the terrain was carried out. This mode requires an additional adjustment (calibration) that involves the dynamics of the measurement at different aperture sizes. With the initial information from the GPS, the calibrated INS measurements and the coordinate conversion, georeferenced points were obtained, and adjusted to centimeters. Whether this georeferencing information depended on the rank of displacements and/or the velocities within the parcel was verified.

On the other hand, in image acquisition control mode, the displacement velocity had to be low, in order to obtain images of the proper resolution. This velocity could not be above 5 cm·s^−1^; yet, under these conditions, it was not possible to measure displacement; it was only possible to identify the beginning and end of movement, as shown in Section 3, with the purpose of editing the acquired videos and to continue with the frame-by-frame analysis, in order to define whether the information is suitable to be processed or whether, on the contrary, it had blurriness problems, or a lack of sharpness.

### 4.1. Calibration and Evaluation of the Proposed INS in Navigation Mode (NM)

#### 4.1.1. Calibration of Navigation Mode

With the aim of determining the functioning of the proposed INS in the displacement measurement within a coffee parcel, different trials were carried out, where aperture size, form of the displacement, total covered space, speed of the tour, and direction of movement were varied. In Table 3, a distribution of points (P) employed to calibrate the measurement of displacements are shown. The distance among each one of the points was considered to be the size of one’s step, which ranged between 0.5 m and 3.75 m. Five types of displacements were generated on the points shown as is illustrated below: (*i*) Movement 1: *P*_00_ a *P*_0i_; (*ii*) movement 2: *P*_00_ a *P*_j0_; (*iii*) movement 3: *P*_00_ a *P*_j0_, *P*_j0_ a *P*_j1_, *P*_j1_ a *P*_01_; (*iv*) movement 4: *P_j_*_1_ a *P*_01_, *P*_01_ a *P*_00_, *P*_00_ a *P*_j0_, and (*v*) movement 5: P_00_ a *P*_j0_, *P*_j0_ a *P*_j1_, *P*_j1_ a *P*_01_, *P*_01_ a *P*_02_, *P*_02_ a *P*_j2_. The GPS coordinates for the start point P_00_ were 1043915.9141 N 831420.7374 E in Gauss-Krüger plane coordinates.

Each ***P*** point was marked on the terrain at different distances in meters (0.5, 0.75, 1, 1.5, 2.25, 3, 3.75 m). For each movement, eight signals were generated with the mobile device and they were processed as detailed in Section 3.

**Figure 7:**
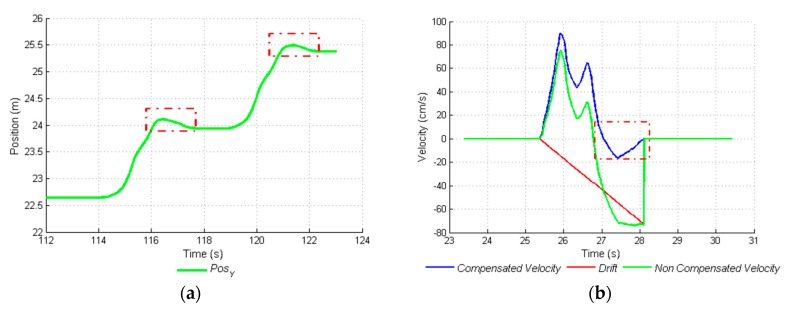
Effect of DRIFT compensation on calculated displacement. (**a**) Negative displacement marked in the boxes in two parts of the vector position; and (**b**) Negative compensated velocity due to the final velocity and DRIFT compensation.

As shown in Figure 7a, the displacement generated by the INS shows small regressions at the end of each step. This is due to the fact that on many occasions, DRIFT compensation generated negative final velocities, as shown in Figure 7b. At no time were there regressions. This situation could have been generated because the median velocity of the movements was 44 cm·s^−1^ (between 45 cm·s^−1^ and 42 cm·s^−1^). At higher velocities, negative DRIFT compensations were not generated. For this reason, an adjustment or calibration was made for the position vector that shows the path. Normally, the path of the parcel was performed at these velocities, due to the complexity of movement in densely vegetated terrain.

In order to calculate the calibration factor of the displacement measurement, a linear regression analysis was performed between the displacement values calculated by the algorithm, and the real values in five signals per movement. The analysis estimated the regression coefficient as a value of 1.4735, a determination coefficient (R^2^) of 0.98, and an intercept equal to zero, according to the t-test at 1%. The value of the regression coefficient of 1.4735 worked as a calibration factor for the values calculated by the algorithm.

**Figure 8:**
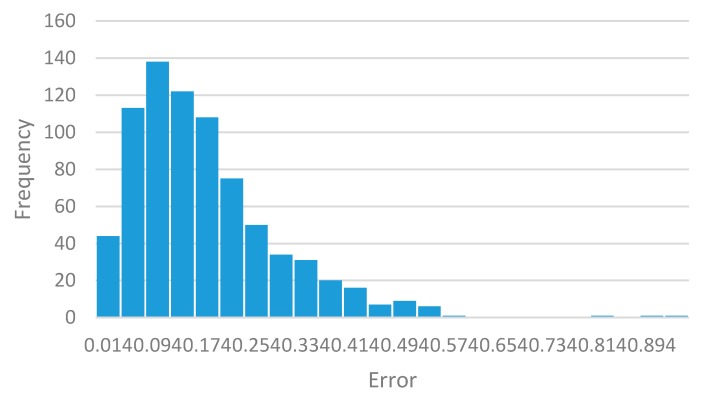
Frequency distribution of the measurement relative error of position with INS.

The system was validated with the three remaining calibrated signals with the regression coefficient found. The functioning of the algorithm was verified by means of a second linear regression analysis between the real displacement values and the measured and adjusted values. The result of this validation showed that the calibrated system had a regression coefficient of 1.0, this indicates that the INS did not overestimate or underestimate displacement values; it estimated them one by one with an R^2^ of 0.98. The mean relative error was found, as the absolute difference between real displacements and calculated displacements. The mean relative error of the measurement in this case was ±0.15 m, for displacements between 0.5 m and 3.75 m, clearly inferior to the one reported for the GPS (from ±2.5 m to ±5 m). The histogram presented in Figure 8 shows the absolute errors of INS and their dynamics.

**Figure 9:**
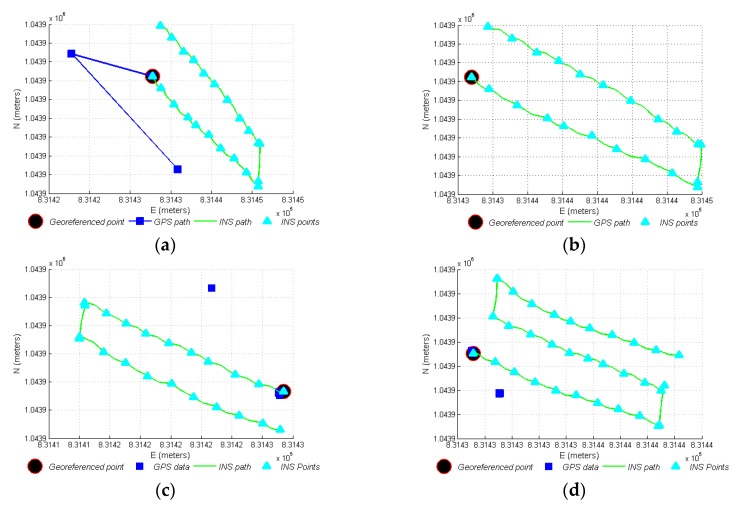
Adjustment and calculation of velocity and position of movement. (**a**) GPS data for movement 3 and path generated by INS; (**b**) Georeferenced data with proposed INS for movement 3 with starting point read from the GPS; (**c**) Georeferenced data with proposed INS for movement 4; (**d**) Georeferenced data with proposed INS for movement 5.

In Figure 9a, points georeferenced with the GPS, out of the real path can be seen; they are considered georeferencing errors. In this case, only four readings were obtained from the GPS, but in fact, 20 referencing points were generated with the INS. Contrarily, in Figure 9b the points georeferenced by means of the proposed INS, which generated a spatial reference modified for movement, with the 20 referenced points and with minimal position errors are shown. In this case, only the first GPS point was taken into account (in Gauss-Krüger plane coordinates NE) as the starting point, with an initial error of 3.18 m, the other georeferencing points were calculated through the displacement information from the INS, and had an error of ±0.15 m. Figure 9c,d show the paths generated by the system in movements 4 and 5, respectively. Use of the proposed INS generated adequate georeferences in terms of the position, sequence, and advance of movement, in different step sizes, velocities, forms of covering spaces, and direction of the INS.

#### 4.1.2. Evaluation of Navigation Mode

**Figure 10:**
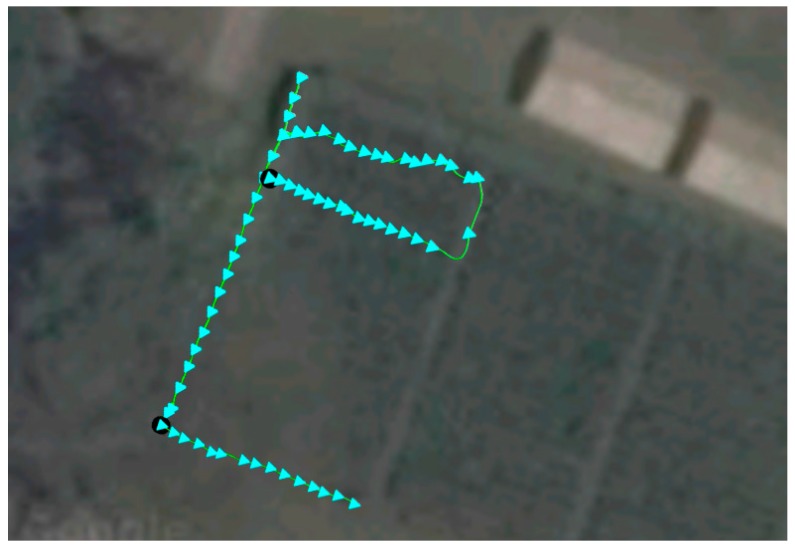
Georeferencing of trees in a coffee-sowed parcel (image taken from Google Maps).

The evaluation of the INS in navigation mode was made on a parcel sown with coffee, with a 1.5 m × 1 m sowing distance. In Figure 10 the results obtained from the parcel sown with coffee, with apertures 1 and 1.5 m long, multiple of the sowing distance, and an initial GPS point of 1,043,868.786 N 831,230.1454 E (with an initial error of 1.8 m over the real value) are shown. Two movement trajectories can be observed: (*i*) one around the edges of the parcel; (*ii*) and a second U-shaped one inside the parcel. Overall, 80 georeferenced points were generated with the INS, whereas the traditional GPS only obtained four points. The evaluation for this case showed that the system neither overestimated nor underestimated displacement and that it was capable of estimating the position of the trees to be checked within the parcel one by one. The displacement velocities were within the range mentioned in Section 4.1.1, from 45 cm·s^−1^ and 42 cm·s^−1^, and the median relative error of the path generated by the INS was ±0.26 m.

### 4.2. Evaluation of the Proposed INS in Image Acquisition Control Mode ACM

When selecting the option for acquiring video/images, the branch to be digitalized was identified and a folder with the name of the branch was created. In this folder, the video and the images were stored as was the text file of the inertial signals of the proposed INS. Once the camera was activated, the branch was positioned as required, the image was focused, and the video recording started. The displacement of the mobile device over the branch started only when the image on the screen was properly focused. The system detected the beginning and end of the displacement and, with this information, the video were edited. With this editing, valid information was acquired for image processing.

**Figure 11:**
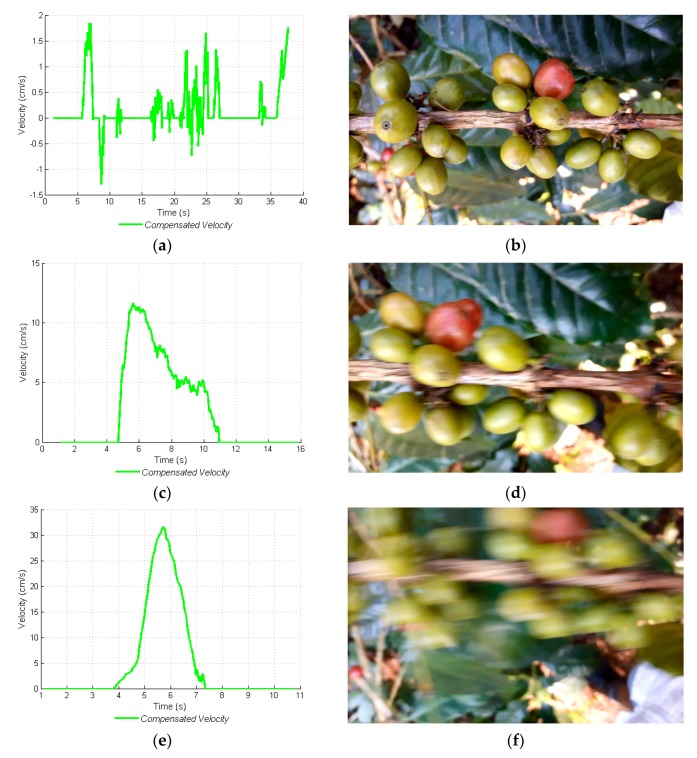
Displacement velocities of the mobile device over the coffee branch. (**a**) Compensated velocity signal. Low velocity; (**b**) Image acquired at low velocity; (**c**) Compensated velocity signal. Medium velocity; (**d**) Image acquired at medium velocity; (**e**) Compensated velocity signal. High velocity; (**f**) Image acquired at high velocity.

The displacement velocity must be such that the images do not have any blurriness or movement-caused interference problems. For this reason, three velocities were determined (low, medium, and high) so as to acquire images and to discover as of what velocity the images or video frames begin to blur. This task was carried out over 50 cm long branches, and the velocity was manually determined, considering the displacement times from the beginning to the end of the branch. The three velocities mentioned are: (*i*) low: below 5 cm·s^−1^; (*ii*) medium: between 5 cm·s^−1^ and 10 cm·s^−1^; and (*iii*) high: above 10 cm·s^−1^. In Figure 11a,c,e the calculated velocity signals with the linear acceleration are shown. Unfortunately, the acquired information for the low velocity (Figure 11a) did not correspond to any movement made, since no backward displacements were generated as shown by the velocity signal, with negative values. With this velocity signal, it was not possible to adequately determine displacement; nonetheless, the acquired images (Figure 11b) were of good quality and did not have any movement-caused blurriness. For medium and high velocities (Figure 11c,e), the information delivered by the linear acceleration was coherent and could be used to measure displacement, but the images it produced were blurry (Figure 11d,f). It was corroborated that, below 5 cm·s^−1^, it was not possible to acquire linear acceleration signals that corresponded to movement and, therefore, displacement could not be measured below that velocity.

**Figure 12:**
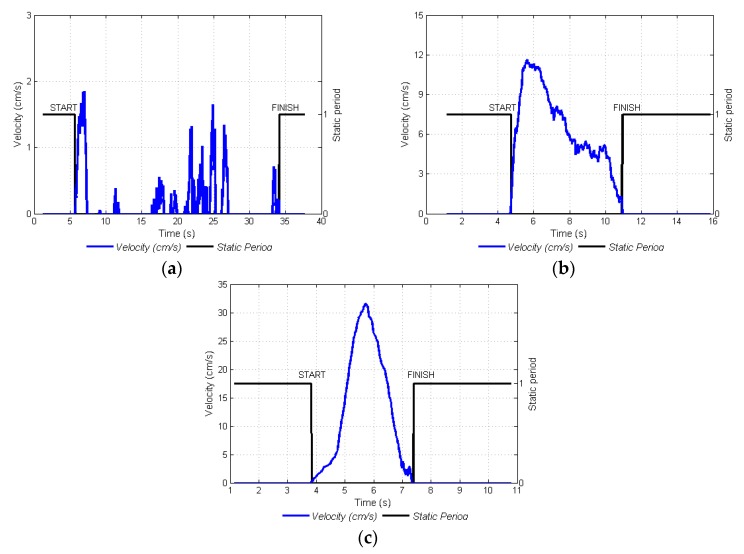
Detection of movement periods for three velocities. (**a**) Low velocity; (**b**) Medium velocity; (**c**) High velocity.

With relation to the type of acquired images, illumination was uncontrolled, flash was not activated, and there was a possibility of poor illumination conditions. For this reason, acquired images may be largely influenced by the absence of light and require the mobile device to move at low velocities in order to acquire adequate images. In this study, it was corroborated that, video frames acquired only at low velocities (Figure 11b), had sharp and coherent information. For medium and high velocities (Figure 11d,f, respectively), the information on glomeruli, fruits, and leaves disappeared due to blurriness. With the aim of finding the start and end of a given video recording, the movement period was defined as the moment when the displacement of the mobile device over the branch started until the moment it ended. In Figure 12a–c, the movement periods for low, medium, and high velocities, can be observed, respectively. With this information, the videos were edited and no frames were taken into account from when the device remained still. The start/end movement times were manually verified and compared with those generated by the proposed navigation system, and a correct identification of the start time was made 99% of the time and of the end time 97% of the time.

### 4.3. Video Acquisition and Image Analysis for Selection Video Frames

A blurriness/sharpness index was determined for the trimmed video frames, in order to define whether they were adequate to be processed or not. In the case that they were adequate, they were selected and the video was shortened to 90 frames for 50 cm long branches. In this subsection the strategy for the calculation of blurriness indexes and the functioning of each one of them for acquired images at different displacement velocities is shown. 

#### Calculation of Blurriness and Sharpness Indexes from Acquired Images at Different Velocities

**Figure 13:**
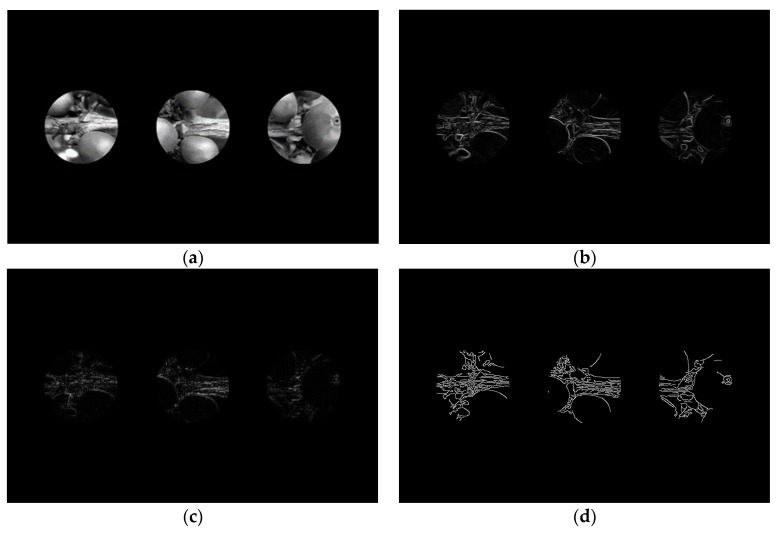
Regions of interest with circular shapes and several blurriness indexes. (**a**) Image in red channel with three ROI; (**b**) Sobel operator on the three ROI; (**c**) Laplacian filter on the three ROI; (**d**) Canny detector on the three ROI.

From each cropped video by movement periods, three frames or images were selected corresponding to the beginning, middle, and end of the movement. In each image, three regions of interest (ROI) with circular shapes were selected in the central zone of the image, located on the right, center, and left side of the image, as shown in Figure 13a. Each ROI had a diameter of 100 pixels, due to their location on the branch, and the location of the coffee fruits in the images. On each region of interest, three edge detectors were applied on the red channel of the original image: (*i*) a Sobel operator with a 3 × 3 kernel in two directions: horizontal (*Sobel.x*) and vertical (*Sobel.y*) (Figure 13b, Equation (9)); (*ii*) a Laplacian filter with 3 × 3 kernel (Figure 13c, Equation (10)); and (*iii*) a Canny detector, capable of adjusting to the lighting conditions in the image, with an inferior limit corresponding to a standard deviation of the complete image (*σ*) in the red channel, and a superior limit of three standard deviations (*3σ*) (Figure 13d, Equation (11)). Regarding the resulting intensity information in each ROI, a sharpness index for each filter was calculated: (*i*) for the Sobel filter, the sum of the square root of the squared *Sobel.x, Sobel.y* values, in each pixel in the ROI was calculated (Equation (9)); (*ii*) for the Laplacian filter, the sum of the absolute value was used (Equation (10)); and (*iii*) for the Canny filter, the sum of the pixels in the ROI was used (Equation (11)):

(9) $$Idx_{sobel}=\sum^{\text{​}}\sqrt{Sobel.x^{2}+Sobel.y^{2}},$$

(10) $$Idx_{laplacian}=\sum^{\text{​}}abs\left(Laplacian\right),$$

(11) $$Idx_{canny}=\sum^{\text{​}}{canny|}_{\sigma }^{3\sigma },$$

Overall, 10 videos, 30 images, and 270 indexes for each one of the three velocities were obtained. Confidence intervals were chosen to be 95%, for each index, region of interest, and mobile device displacement velocity.

**Figure 14:**
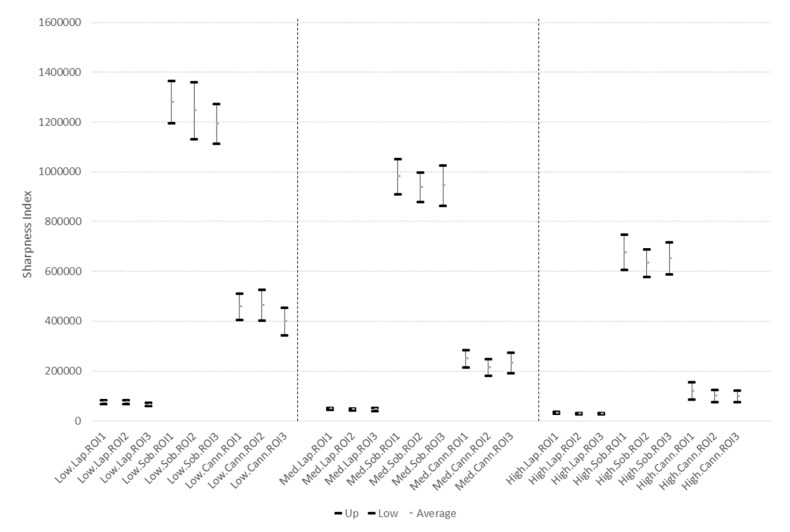
Confidence intervals for sharpness index at different velocities of image acquisition.

In Figure 13 the confidence intervals for each index, in the three velocities, the three edge detectors, and the three regions of interest are shown. On the axis of the *X* set identifiers of *velocity.index.ROI* are observed. This identifier shows: (*i*) *velocity* with three values Low, Medium and High; (*ii*) *index* with three options Sobel, Laplacian and Canny; (*iii*) ROI three regions of interest ROI1, ROI2, and ROI3. Figure 14 shows that there is no statistical differentiation between blurriness indexes in the three ROI, and that the three blurriness indexes are statistically different for the same velocity. Additionally, a single blurriness index is statistically different at the three velocities. For this reason, it is possible to infer that the blurriness indexes are related to the velocity of acquisition of an image. The blurriness index of a single ROI is sufficient to determine that of the other two ROI. Any of the three filters can be used to determine image clarity, since each one shows statistical differentiation at different velocities. A second aspect, in order to find the filter of choice, is that of the computational cost of implementation on the mobile device.

**Figure 15:**
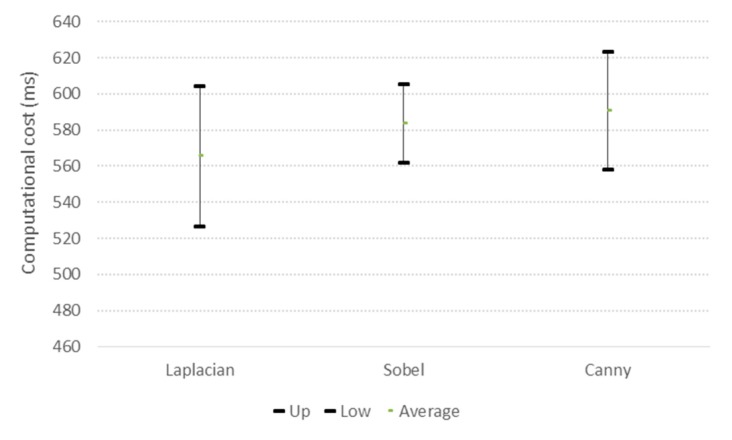
Confidence intervals for the computational cost of each applied index/filter to calculate sharpness.

In Figure 15, confidence intervals are shown for the computational cost of calculation of each index for the ROI in the three selected images for each video, the computational cost is determined in the mobile device. It was concluded that there was no statistical difference in the computational cost between one index and another.

Under the two selection criteria for the blurriness rate (statistical difference for each velocity and minimum computational requirement) either of the indexes work adequately. Analyzing the results obtained with the confidence intervals in Figure 14, only three types of video frames were accepted: those that present, in one of the ROI, a sharpness factor above 1,100,000 for the Sobel filter and index, above 54,000 for the Laplacian filter and index, or above 290,000 for the Canny filter and index. If the value was below these values, the image was considered to not have the necessary sharpness to be processed, because it could have been acquired at velocities above 5 cm·s^−1^, which generates blurriness in the video frames.

### 4.4. Mobile Application Interface for Navigation and Image Acquisition on Coffee Plantations

**Figure 16:**
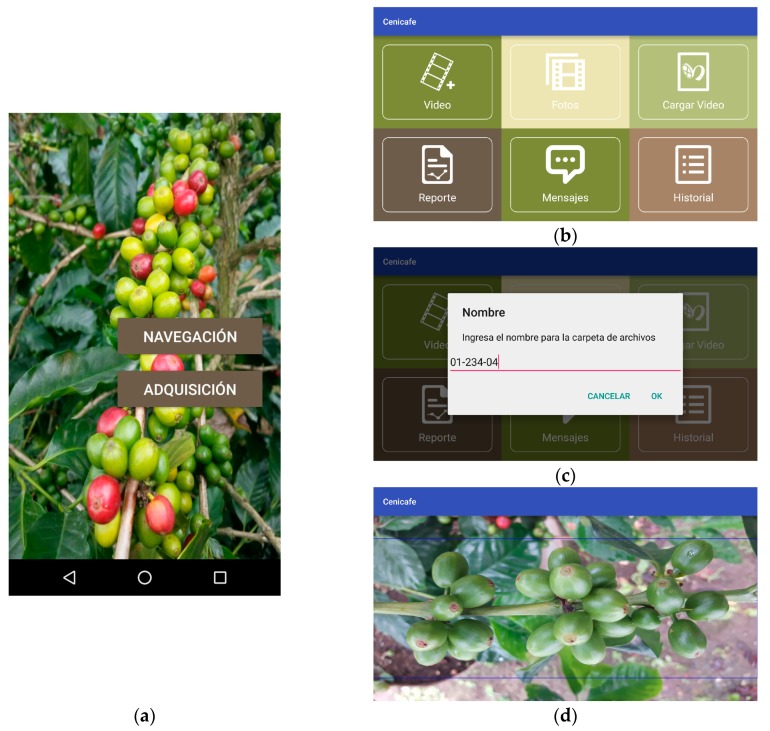
Appearance of the application. (**a**) Initial screenshot of the developed app; (**b**) Menu of the ACM mode; (**c**) Registration of the branch to be acquired; (**d**) Focus zone of the camera on the branch.

The developments described in the previous sections were implemented on a mobile device. The appearance of the application is shown in Figure 16. In Figure 16a, the initial screenshot, where it is possible the select the operating mode, is shown. When selecting Navigation mode, the menu does not change its look and the system stores the inertial information to be processed. When selecting Acquisition mode, the menu of Figure 16b is displayed. With this configuration, a new video can be recorded, static images can be taken, previous videos can be loaded, or reports from previous acquisitions can be seen. Should one decide to record a video or to take an image, the system shows the screenshot in Figure 16c, in which the folder is named with the identifier of each branch and, afterwards, the screenshot in Figure 16d is shown. After, one can have a preview of the mobile device’s camera, where the focus and beginning of video recording or the capture of images can be controlled.

The application, generated in Android Studio, uses OpenCV for image training, and to make filtering calculations. It has two acquisition modes (images or video). Moreover, it uses the inertial sensors and the device’s GPS to georeference each acquired image. The application is compiled for devices running Android 4.4+ versions and uses libraries such as SupportDesign, Retrofit, and AndroidAnnotations for its functioning.

## 5. Conclusions 

The movement of a mobile device was accurately identified as it recorded videos and moved over the branches of a coffee tree. This technological characteristic caused the resulting videos of a sampling to have smaller sizes, and causes less space to be required on servers or the device itself. Additionally, the type of blurriness in an image might indicate the velocity with which the video was recorded, since this cannot be measured when the displacement is made at velocities below 5 cm·s^−1^. 

The inertial navigation system developed uses sensors available on the mobile device, and achieves locations relative to ±0.15 m, with less error than a GPS. The GPS gives the initial value for the path, and may reflect error between ±2.5 m and ±5 m. This initial error directly affects the path’s total error, which can be ±5.15 m. However, despite the system’s total error, the INS guarantees that there is no loss of information from the path of a parcel, as can happen when using the mobile device’s GPS exclusively. This is a notable advantage for the tool developed, since for densely planted crops such as coffee, obtaining information between trees planted centimeters apart is of vital importance. Each tree can be georeferenced with the correct locations on parcels, and with the spatially referenced information, it is possible to generate logistical strategies for farm work, in a selective manner on each parcel, in the future. In hopes of solving the problem of precision with the GPS, in future investigations, a starting point could be generated with the GoogleMaps interface, creating a location marker on the parcels to be checked.

With relation to other developments, this study hoped to integrate the acquisition control of moving images, their spatial location, and the quality of the information, so as to have an economical, precise, and versatile tool for acquiring information about coffee branches, by means of the integration of multiple sensors on a mobile device. The tool described in this article shows a solution which could be applied not only to coffee farms, but also to other crops, such as apples, oranges, etc.

## Figures and Tables

**Table 1:** Sensors incorporated in the mobile device.

Sensor	Reference	Axes
**Accelerometer**	MPU6500	3
**Gyroscope**	MPU6500	3
**Magnetometer**	AK09911C	3
**GPS**	-	-

**Table 2:** Constants for the calculation of local maxima (*K*) and minimum threshold value (*min(THDis)*).

Constants	Linear Acceleration *X*	Linear Acceleration *Y*	Angular Velocity *Y*
***K***	1.8	0.44	1.8
***min(***$THD_{is}$***)***	0.15	0.15	0.05

**Table 3:** Points employed for the adjustment in navigation mode.

$P_{00}$	$P_{01}$	$P_{02}$	…	$P_{0i}$
$P_{10}$	$P_{11}$	$P_{12}$	…	$P_{1i}$
$P_{20}$	$P_{21}$	$P_{22}$	…	$P_{2i}$
⋮	⋮	⋮	⋱	⋮
$P_{j0}$	$P_{j1}$	$P_{j2}$	…	$P_{ji}$
